# Dry eye disease symptoms and associated risk factors in a Norwegian clinical cohort

**DOI:** 10.1111/aos.70143

**Published:** 2026-04-11

**Authors:** Mazyar Yazdani, Fredrik A. Fineide, Reza A. Badian, Ayyad Zartasht Khan, Helge Rootwelt, Jan Terje Kvaløy, Tor Paaske Utheim

**Affiliations:** ^1^ Department of Medical Biochemistry Oslo University Hospital, Rikshospitalet Oslo Norway; ^2^ Department of Plastic and Reconstructive Surgery Oslo University Hospital Oslo Norway; ^3^ The Norwegian Dry Eye Clinic Oslo Norway; ^4^ Department of Computer Science Oslo Metropolitan University Oslo Norway; ^5^ Department of Ophthalmology Østfold Hospital Trust Moss Norway; ^6^ Department of Medical Biochemistry Oslo University Hospital Ullevål, Oslo Norway; ^7^ Department of Ophthalmology Sørlandet Hospital Arendal Arendal Norway; ^8^ Department of Mathematics and Physics University of Stavanger Stavanger Norway; ^9^ Department of Ophthalmology Oslo University Hospital Oslo Norway; ^10^ Institute of Clinical Medicine, Faculty of Medicine University of Oslo Oslo Norway; ^11^ Department of Ophthalmology Stavanger University Hospital Oslo Norway; ^12^ Department of Ophthalmology Vestre Viken Hospital Trust Drammen Norway; ^13^ Department of Ophthalmology Vestfold Hospital Trust Tønsberg Norway; ^14^ Department of Quality and Health Technology, the Faculty of Health Sciences University of Stavanger Stavanger Norway; ^15^ Department of Oral Biology, Faculty of Dentistry University of Oslo Oslo Norway; ^16^ National Centre for Optics, Vision and Eye Care, Department of Optometry, Radiography and Lighting Design, Faculty of Health Sciences University of South‐Eastern Norway Kongsberg Norway; ^17^ Department of Health and Nursing Science, the Faculty of Health and Sport Sciences University of Agder Grimstad Norway; ^18^ Department of Ophthalmology, Faculty of Life Course Sciences and Medicine King's College London London UK; ^19^ Department of Research and Development Oslo Metropolitan University Oslo Norway

**Keywords:** age, allergy, contact lens wear, DEQ‐5, diet, diurnal variation, dry eye disease, medications, omega‐3, OSDI, risk factors, sex, time of day

## Abstract

**Background:**

The relationship between dry eye disease (DED) symptoms and different demographic, clinical and lifestyle factors remains poorly understood. Norway's cold climate and dry indoor air may accelerate tear evaporation, contributing to a very high meibomian gland dysfunction prevalence, as found in multiple studies. This suggests that environmental factors and age converge to drive evaporative dry eye as a near‐universal finding in the Norwegian population. This study aimed to assess demographic, clinical and lifestyle correlates of DED symptoms, with a focus on sex‐related differences and diurnal variation.

**Methods:**

Between September 2021 and December 2023, 1044 individuals (303 males, 741 females) with symptoms of DED were enrolled at the Norwegian Dry Eye Clinic. All participants completed the Ocular Surface Disease Index (OSDI) and the Dry Eye Questionnaire‐5 (DEQ‐5) questionnaires, as well as an in‐house, un‐validated questionnaire. Clinical examinations and history‐taking were conducted in a standardized manner by two experts. Associations between questionnaire scores and variables were analysed using Chi‐squared tests, Kruskal–Wallis tests, Spearman correlations and logistic regression.

**Results:**

Symptomatic DED above clinical cut‐off levels was more prevalent in females than in males. Symptom severity was lowest in the late morning, and significant differences in both OSDI and DEQ5 scores were observed between individuals with and without reported omega‐3 supplementation (with slightly higher scores in those taking omega‐3). Allergies were associated with time‐of‐day symptom severity. Multivariable logistic regression revealed female sex, age below 65, not wearing contact lenses and refractive lens exchange surgery as significant risk factors for reporting more severe symptoms among the individuals in the database.

**Conclusions:**

Severity of dry eye symptoms in this symptomatic, clinic‐based population was significantly associated with several demographic and clinical factors. Incorporating standardized timing of assessments may enhance diagnostic accuracy in both research and clinical settings for DED.

## 1 INTRODUCTION

Dry eye disease (DED) is a prevalent, multifactorial condition of the ocular surface characterized by loss of tear film homeostasis, leading to symptoms of discomfort, visual disturbance and potential damage to the ocular surface (Wolffsohn et al., 2025). The disease affects a significant proportion of the population worldwide and is increasingly recognized as a public health concern due to its impact on quality of life, productivity and healthcare burden. While DED can occur at any age, its prevalence increases with senescence. It is consistently reported to be higher among females, suggesting the influence of both hormonal and other biological factors in disease susceptibility and symptomatology (Craig et al., 2017; Stapleton et al., 2025; Sullivan et al., 2017).

The clinical presentation of DED is heterogeneous, with symptoms often fluctuating throughout the day, being influenced by numerous intrinsic and extrinsic factors. Environmental exposures, systemic comorbidities, medications, dietary intake and lifestyle are all considered potential modulators of dry eye symptoms (Britten‐Jones et al., 2024). Among these, omega‐3 fatty acid supplementation has attracted interest due to its anti‐inflammatory properties and potential protective role in ocular surface health (Britten‐Jones et al., 2024). Likewise, the presence of allergic conditions (Villani et al., 2018), underlying endocrine disorders such as hypothyroidism (Gorimanipalli et al., 2023), and specific dietary patterns may contribute to symptom severity (Signes‐Soler & Javaloy Estañ, 2019), along with a range of other internal and external factors. However, comprehensive investigations into how these variables interact with subjective symptom burden, especially over the course of the day, remain limited.

Despite growing evidence, most studies have focused either on population‐based cohorts (e.g., Vehof et al., 2021) or experimental interventions (e.g., Morthen et al., 2021), leaving a gap in our understanding of how diverse clinical and lifestyle factors jointly influence symptom severity in patients who actively seek care for DED. Moreover, few investigations have systematically explored how symptom burden evolves over the course of a typical day (e.g., Schindelar et al., 2008), an aspect that is highly relevant to clinical decision‐making.

Validated symptom‐based tools, such as the Ocular Surface Disease Index (OSDI) and Dry Eye Questionnaire‐5 (DEQ5), have become essential in both clinical and research settings for quantifying patient‐reported symptoms and assessing the impact of DED (Wolffsohn et al., 2017). Nevertheless, standardized questionnaires may not fully capture the nuanced variability in symptomatology across the day or provide insight into coexisting systemic or lifestyle‐related influences. Incorporating an in‐house questionnaire tailored to local clinical practise could complement existing procedures and enhance the detection and characterization of symptoms. However, using such a questionnaire has its shortcomings.

Norway has a generally cold climate, and indoor heating including the use of air‐to‐air heat pumps can cause dry indoor air quality, which may exacerbate DED by accelerating tear evaporation. Two studies based on Norwegian cohorts demonstrated a high prevalence of meibomian gland dysfunction (MGD) in both a symptomatic clinic‐based population (93.8%) (Badian et al., 2021) and a randomly selected general population cohort of 65‐year olds (90.5%) (Badian et al., 2026). In the former, the majority of first‐time clinic visitors had elevated OSDI scores (≥13), yet 87.8% of those with normal OSDI scores (<13) had MGD. The latter study showed that 20.4% of the general population cohort met the diagnostic criteria for DED, with MGD present in 100% of those diagnosed with DED. However, the extent to which symptom severity reporting is shaped by demographic factors, systemic comorbidities, medications, dietary habits, gender differences in symptom‐reporting, and day‐to‐day variation remains unknown. Addressing this gap is important for refining risk stratification and developing more individualized care strategies. Hence, in this study, we focused on the roles of sex, age, omega‐3 supplementation, dietary patterns, allergy, time‐of‐day variability, medical conditions and current medication use. By combining validated assessment tools with our detailed, yet un‐validated 21‐item in‐house questionnaires, we sought to characterize symptom patterns and identify modifiable and non‐modifiable risk factors that may facilitate personalized approaches to the diagnosis and management of DED.

## 2 MATERIALS AND METHODS

### 2.1 Patients

Data from the first‐time consultation of patients included in the Norwegian Dry Eye Cohort 2023 (NDEC2023) were used. NDEC2023 is a specialized, prospective observational cohort established at the Norwegian Dry Eye Clinic to investigate the pathophysiology, clinical features and treatment responses in patients diagnosed with DED. The cohort comprises participants (*n* = 1044) referred or self‐referred to the clinic due to dry eye‐related symptoms and signs, including those with suspected or confirmed DED of various etiologies. NDEC2023 incorporates a standardized diagnostic protocol that encompasses the symptoms and signs of DED. Participants were enrolled consecutively between September 2021 and December 2023, with plans for long‐term follow‐up to study disease progression and therapeutic outcomes. No calculation of sample size was performed, and no inclusion or exclusion criteria were applied beyond the ability to provide written, informed consent. Participants were recruited when presenting at the clinic. The study was approved by the Regional Ethics Committee of South‐East Norway (reference ID 6892) and adhered to the Declaration of Helsinki.

### 2.2 Clinical evaluation

Following signed informed consent and prior to clinical examination, all participants were given paper copies of and completed the OSDI (cut‐off value: 13 [Wolffsohn et al., 2017]), DEQ5 (cut‐off value: 6 [Rodrigues et al., 2021]), as well as an un‐validated in‐house questionnaire containing information regarding comorbidities, current medications, diet and the presence of various symptoms at different times of the day (scored 0–10 with 10 being the most pronounced). Symptom ratings were based on recall. Time‐of‐day symptom severity was intended to be recorded separately for each eye. However, due to the design of the questionnaire, participants interpreted the item as referring to both eyes and completed only the rightmost column. Consequently, data from a single eye were included in the analysis. A detailed general and ophthalmological medical history was also gathered in a standardized manner. Age 65 was chosen as the cut‐off value for the older age groups as it is considered the threshold for the age category known as young‐old, which starts at age ≥ 65 (Badian et al., 2026; Chung et al., 2023). Two dry eye expert clinicians (Fredrik A. Fineide and Tor P. Utheim) were responsible for conducting all interviews and examinations of all participants.

### 2.3 Statistical analysis

Continuous variables were summarized by means, standard deviations, medians and quartiles, as appropriate, and categorical variables by numbers and proportions. Missing values were not imputed and were treated as such. The use of omega‐3 was indicated by ticking a box in the questionnaires. Every individual who ticked the box was registered as a user. Similarly, participants who did not select a specific diet were assumed to follow a regular diet. Information regarding dosage, formulation and duration of supplementation was not recorded; diet and supplementation use were noted in a strictly binary fashion.

To test for differences in distribution between groups, the Kruskal–Wallis test was used for continuous variables, and the Chi‐squared test was used for categorical variables. Spearman correlations were used to assess associations between continuous variables. Logistic regressions were applied to evaluate associations between an OSDI score above or equal to the clinical cut‐off value of 13 and potential risk factors. Both univariable and multivariable logistic regression models were performed to examine the independent and adjusted effects of each risk factor. An exploratory backward stepwise selection was also conducted. Nagelkerke pseudo‐*R* ^2^ was reported for the multivariable models and co‐linearity among predictor variables was assessed by cross‐tabulations. Due to the explorative nature of the study, statistical test outcomes should be interpreted as exploratory. Tests with a *p*‐value < 0.05 were considered significant; however, given the large number of tests conducted, the tables of results also included significance thresholds adjusted using per‐table Bonferroni corrections for multiple testing. The statistical analyses were performed in R, version 4.3.3 (R Core Team, 2024).

## 3 RESULTS

Table 1 presents the demographic distribution of dry eye symptoms based on OSDI and DEQ5 questionnaire results as well as missing data distributed by age. A significant sex disparity was observed, with a higher prevalence of DED symptoms above clinical cut‐off values among females (OSDI: 87.3%, DEQ5: 89.9%) compared to males (OSDI: 77.9%, DEQ5: 87.1%), showing statistical significance in both assessments (OSDI: *p* < 0.001, DEQ5: *p* = 0.003). Age‐wise, DED symptoms above clinical cut‐off values were equally frequent in all age groups, peaking in the 20–39 (OSDI: 87.7% and DEQ5: 91.7%) and 40–59 (OSDI: 88.0% and DEQ5: 91.6%) age ranges. There was no statistically significant difference in the age distribution between individuals with symptom scores above versus below the cut‐off (*p* > 0.05). These findings reinforce the role of sex in DED symptom severity reporting.

**TABLE 1:** Demographic factors (age and sex) associated with dry eye disease severity among patients according to OSDI and DEQ5 questionnaires.

OSDI		Dry eye Disease^a^			*p*‐value^b^
		Below cut‐off value (<13)	Above cut‐off value (≥13)	Missing	
		*N* (%) [M, F]	*N* (%) [M, F]	*N* (%)	
Age	0–19	2 (20.0%) [1, 1]	7 (70.0%) [1, 6]	1 (10.0%)	(OSDI vs. age) 0.099
	20–39	24 (9.5%) [12, 12]	222 (87.7%) [80, 142]	7 (2.8%)	
	40–59	34 (8.7%) [17, 17]	344 (88.0%) [67, 277]	13 (3.3%)	
	60–79	49 (13.9%) [20, 29]	281 (79.8%) [81, 200]	22 (6.2%)	
	80–99	4 (10.5%) [4, 0]	29, (76.3%) [7, 22]	5 (13.2%)	
Sex	Female	59 (8.0%)	647 (87.3%)	35 (4.7%)	(OSDI vs. Sex) <0.001*
	Male	54 (17.8%)	236 (77.9%)	13 (4.3%)	

DEQ5		Dry eye Disease^a^			*p*‐value^b^
		Below cut‐off value (<6)	Above cut‐off value (≥6)	Missing	
		*N* (%) [M, F]	*N* (%) [M, F]	*N* (%)	
Age	0–19	1 (10.0%) [1, 0]	8 (80.0%) [0, 8]	1 (10.0%)	(DEQ5 vs. age) 0.349
	20–39	15 (5.9%) [9, 6]	232 (91.7%) [85, 147]	6 (2.4%)	
	40–59	13 (3.3%) [6, 7]	358 (91.6%) [78, 280]	20 (5.1%)	
	60–79	19 (5.4%) [8, 11]	301 (85.5%) [91, 210]	32 (9.1%)	
	80–99	3 (7.9%) [1, 2]	31 (81.6%) [10, 21]	4 (10.5%)	
Sex	Female	26 (3.5%)	666 (89.9%)	49 (6.6%)	(DEQ5 vs. Sex) 0.003*
	Male	25 (8.3%)	264 (87.1%)	14 (4.6%)	

Abbreviations: %, percentage; DEQ5, Five‐item dry eye questionnaire; F, female; M, male; *N*, sample size; OSDI, ocular surface disease index.

^a^ The cut‐off values are based on recommendations from the Tear Film and Ocular Surface Society (TFOS) Dry Eye Workshop (DEWS) II (Wolffsohn et al., 2017). Percentages are row‐wise percentages.

^b^ *p*‐values <0.05 are shown in bold; those remaining significant after Bonferroni correction are marked with an asterisk (*).

How the patient‐graded symptom severity varied during the day is reported in Figure 1 and Table 2. The severity score has a notable skew distribution with a large number of 0's (Figure S1) implying the lower whiskers at the boxplots are at the lower end of the box. The severity changes significantly during the day, although not with a large difference between the time points, with the highest severity reported in the morning and evening, and lowest at late morning.

**FIGURE 1:**
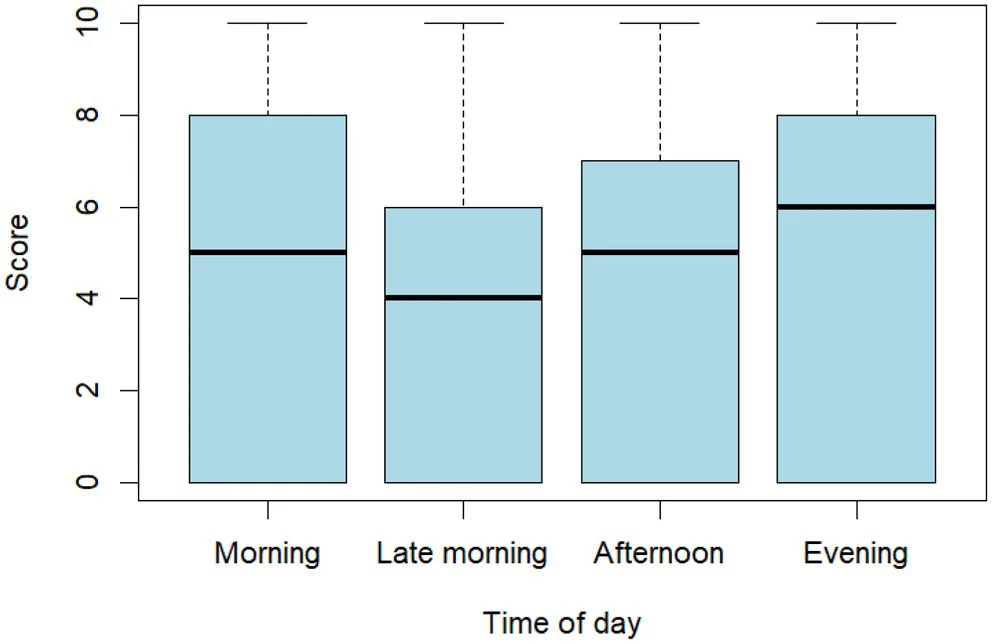
Box‐plots of the time‐of‐day variables. There is a significant difference in score distribution over the day (*p* < 0.001; see Table 2 for details).

**TABLE 2:** Patient‐graded time‐of‐day severity.

		Time of day^a^			
		Morning (*n* = 1044)	Late morning (*n* = 1044)	Afternoon (*n* = 1044)	Evening (*n* = 1044)
Median (quartiles)		5 (0–8)	4 (0–6)	5 (0–7)	6 (0–8)
Test for difference^b^	Late morning	<0.0001*			
	Afternoon	**0.032**	<0.0001*		
	Evening	0.101	<0.0001*	<0.0001*	

^a^ Time of day was categorized as morning (before 09:00), late morning (09:00–12:00), afternoon (12:00–18:00) and evening (after 18:00).

^b^ *p*‐values <0.05 are shown in bold; those remaining significant after Bonferroni correction are marked with an asterisk (*).

Symptoms correlation to the severity at different times of the day was assessed using Spearman correlation (Table S1). Gritty sensation, light sensitivity and blurry vision, as measured by our in‐house questionnaire, showed increasing associations with time of day, with the strongest associations observed for gritty sensation and the evening score (0.33 for OD and 0.36 for OS). Notably, the correlation values for the left and right eyes were closely aligned. All correlations were significant (*p* < 0.001), except for the non‐significant result observed for excessive tearing in the right eye during the evening (*p* = 0.062). These findings highlight the importance of accounting for diurnal symptom variability when assessing dry eye severity and treatment effectiveness.

Spearman correlation analysis was conducted to evaluate the relationship between dry eye diagnostic questionnaires (OSDI and DEQ5) and patient‐graded symptom severity at different times of the day (Table 3). Both OSDI and DEQ5 correlated positively with time‐of‐day symptom scores (OSDI: *r* = 0.19–0.35; DEQ5: *r* = 0.19–0.37), with the strongest correlations with the evening score. Notably, all correlations were statistically significant (*p* < 0.001). These results highlight the importance of considering time‐of‐day effects in DED assessment.

**TABLE 3:** Correlations between dry eye diagnostic questionnaires (OSDI and DEQ5) and patient‐graded time‐of‐day severity (Spearman correlation).

Spearman's rho		OSDI (*n* = 996)	DEQ5 (*n* = 981)	Time of day^a^			
				Morning (*n* = 1044)	Late morning (*n* = 1044)	Afternoon (*n* = 1044)	Evening (*n* = 1044)
OSDI		1.00					
DEQ5		0.67	1.00				
Time of day	Morning	0.19	0.19	1.00			
	Late morning	0.33	0.28	0.67	1.00		
	Afternoon	0.34	0.33	0.58	0.85	1.00	
	Evening	0.35	0.37	0.60	0.70	0.79	1.00

*Note*: All correlations were significant with *p* < 0.001 and remained significant after Bonferroni correction.

Abbreviations: DEQ5, Five‐item dry eye questionnaire; OSDI, ocular surface disease index; rho, Spearman's rank correlation coefficient.

^a^ Time of day was categorized as morning (before 09:00), late morning (09:00–12:00), afternoon (12:00–18:00) and evening (after 18:00).

Associations between omega‐3 supplementation, dietary patterns, allergy and dry eye symptom severity (OSDI and DEQ5 scores) were evaluated using the Kruskal–Wallis test (Table 4). Significant differences in OSDI and DEQ5 scores were observed between individuals with and without reported omega‐3 supplementation (*p* = 0.007 and *p* = 0.006, respectively), with slightly higher scores in those reporting taking omega‐3 supplements. The presence of allergy showed no significant association with DED symptoms, as measured by OSDI (*p* = 0.141) or DEQ5 (*p* = 0.095). However, when considering the time of day, individuals with allergies reported significantly higher symptom severity in the morning (*p* < 0.0001), late morning (*p* = 0.026), afternoon (*p* = 0.008) and evening (*p* = 0.013). Among dietary patterns, no significant difference in OSDI and DEQ5 scores was found. Considering the time of day, differences between diet groups reached statistical significance only in the morning (*p* = 0.004) and late morning (*p* = 0.038) groups, with pescatarians showing the highest mean values. Note that most of these associations are not significant after a strict Bonferroni correction and should thus be interpreted with caution (Table 4).

**TABLE 4:** Associations between dry eye diagnostic questionnaires (OSDI and DEQ5) and other factors with categorical variables, including omega‐3 supplementation, allergy presence and diet^a^ (Kruskal–Wallis test).

		Omega‐3 supplementation			Allergy			Diet				
		Yes	No	*p* ^c^	Yes	No	*p* ^c^	Vegan	Vegetarian	Pescatarian	Other	*p* ^c^
		*N* (missing)	*N* (missing)		*N* (missing)	*N* (missing)		*N* (missing)	*N* (missing)	*N* (missing)	*N* (missing)	
		Median (IQR)	Median (IQR)		Median (IQR)	Median (IQR)		Median (IQR)	Median (IQR)	Median (IQR)	Median (IQR)	
		Mean ± SD	Mean ± SD		Mean ± SD	Mean ± SD		Mean ± SD	Mean ± SD	Mean ± SD	Mean ± SD	
OSDI		490 (24) 40 (22, 57) 40.92 ± 22.37	506 (24) 36 (19, 52) 37.35 ± 22.41	**0.007**	293 (8) 39 (23, 56) 40.77 ± 22.12	703 (40) 38 (20, 54) 38.42 ± 22.57	0.141	10 (1) 32.00 (23, 38) 38.70 ± 26.39	11 (0) 25 (14.50, 45) 30.18 ± 20.55	16 (1) 48 (37, 61) 47.94 ± 20.06	959 (46) 38 (20, 55) 39.07 ± 22.45	0.200
DEQ5		483 (31) 14 (11, 16) 13.24 ± 4.22	498 (32) 13 (9, 16) 12.47 ± 4.33	**0.006**	287 (14) 14 (11, 16) 13.25 ± 4.20	694 (49) 13 (10, 16) 12.68 ± 4.32	0.095	10 (1) 11.50 (9, 12) 11.20 ± 5.43	11 (0) 13 (8, 14.50) 10.91 ± 4.83	16 (1) 14.50 (10, 17) 14.06 ± 4.57	944 (61) 13 (10, 16) 12.87 ± 4.26	0.248
Time of day^b^	Morning	514 (0) 5 (0, 8) 4.57 ± 3.84	530 (0) 5 (0, 8) 4.45 ± 3.84	0.613	301 (0) 6 (1, 9) 5.34 ± 3.85	743 (0) 4 (0, 8) 4.17 ± 3.78	<0.001*	11 (0) 6 (0, 8) 4.64 ± 4.08	11 (0) 8 (6, 8.50) 6.82 ± 2.60	17 (0) 8 (7, 9) 7.41 ± 3.04	1005 (0) 5 (0, 8) 4.43 ± 3.84	**0.004**
	Late morning	514 (0) 4 (0, 6) 3.73 ± 3.30	530 (0) 3 (0, 6) 3.57 ± 3.33	0.419	301 (0) 4 (0, 7) 3.98 ± 3.33	743 (0) 4 (0, 6) 3.51 ± 3.30	**0.026**	11 (0) 6.00 (1.50, 6.50) 4.55 ± 3.30	11 (0) 4 (3.50, 7) 4.91 ± 2.55	17 (0) 6 (5, 8) 5.53 ± 3.47	1005 (0) 3 (0, 6) 3.59 ± 3.31	**0.038**
	Afternoon	514 (0) 5 (0, 7) 4.41 ± 3.47	530 (0) 4 (0, 7) 4.07 ± 3.49	0.126	301 (0) 5 (0, 8) 4.70 ± 3.46	743 (0) 5 (0, 7) 4.05 ± 3.48	**0.008**	11 (0) 4 (0, 5) 3.64 ± 3.59	11 (0) 5 (3.50, 6.50) 5.00 ± 2.32	17 (0) 7 (4, 8) 5.71 ± 3.57	1005 (0) 5 (0, 7) 4.21 ± 3.49	0.246
	Evening	514 (0) 6 (0, 8) 5.01 ± 3.76	530 (0) 5 (0, 8) 4.72 ± 3.67	0.204	301 (0) 6 (1, 8) 5.32 ± 3.65	743 (0) 5 (0, 8) 4.67 ± 3.73	**0.013**	11 (0) 4 (2, 6.50) 4.55 ± 3.56	11 (0) 7 (5, 8.50) 6.82 ± 2.14	17 (0) 8 (5, 9) 6.53 ± 3.43	1005 (0) 5 (0, 8) 4.81 ± 3.73	0.100

Abbreviations: DEQ5, Five‐item dry eye questionnaire; IQR, interquartile range; *N*, sample size; OSDI, ocular surface disease index; SD, standard deviation.

^a^ The diet has four categories.

^b^ Time of day was categorized as morning (before 09:00), late morning (09:00–12:00), afternoon (12:00–18:00) and evening (after 18:00).

^c^ The *p*‐values are calculated using the Kruskal–Wallis test for differences in distribution. *p*‐values <0.05 are shown in bold; those remaining significant after Bonferroni correction are marked with an asterisk (*).

We further explored associations between these parameters using the Kruskal–Wallis test (omega‐3 supplementation, dietary patterns and allergy) and whether they were related to specific dry eye symptoms (Table S2). For the right eye (Oculus Dexter; OD), omega‐3 supplementation was significantly associated with gritty sensation (*p* = 0.006), burning sensation (*p* = 0.021), light sensitivity (*p* = 0.009), stinging sensation (*p* = 0.033) and blurry vision (*p* = 0.012). The presence of allergy showed significant associations with red eyes (*p* = 0.039) and itching (*p* = 0.011). For the left eye (Oculus Sinister; OS), omega‐3 supplementation was significantly associated with gritty sensation (*p* = 0.026), light sensitivity (*p* = 0.008) and blurry vision (*p* = 0.013), while the presence of allergy was linked to blurry vision (*p* = 0.040), red eye (*p* = 0.027) and itching (*p* = 0.019). The diet had fewer significant effects, with a notable association observed in both eyes, specifically a burning sensation (*p* = 0.037 for OD and *p* = 0.014 for OS). These findings are consistent with the observed associations between allergy and specific symptom patterns. Additionally, while dietary patterns showed some associations, their impact on dry eye symptoms remains less clear and requires further investigation. Only 3% of the participants reported following a special diet: 16 pescatarians, 11 vegetarians and 10 vegans. There was no significant association when a strict Bonferroni correction was applied.

A binary logistic regression analysis was conducted to identify factors associated with an increased likelihood of exceeding the OSDI cut‐off score (≥13). The results are presented in Table 5 and Figure 2. First, considering the univariable analyses, when age ≥ 65 was used as the threshold for older age, it was associated with a significant reduction (odds ratio [OR] = 0.63, *p* = 0.030) in self‐reported symptom severity. Similarly, contact lens wear was independently associated with reduced odds of exceeding the cut‐off (OR = 0.59, *p* < 0.001). Sex emerged as a significant risk factor with an OR of 2.51 (*p* < 0.001) for females versus males. Among medical conditions, hypothyroidism was the only significant risk factor (OR = 3.13, *p* = 0.028), and this is not significant after Bonferroni correction.

**TABLE 5:** Binary logistic regression showing associations with higher symptom burden using OSDI score above the cut‐off value (≥13).

Risk Factors^a^	Univariable analyses		Multivariable analysis, all^b^		Multivariable analysis, selection^c^	
	OR (95% CI)	*p* ^d^	OR (95% CI)	*p* ^d^	OR (95% CI)	*p* ^d^
Age (≥65)^e^	0.63 (0.42–0.95)	**0.030**	0.46 (0.29–0.73)	0.001*	0.48 (0.31–0.76)	0.002*
Sex (female vs. male)	2.51 (1.68–3.73)	<0.001*	2.35 (1.55–3.54)	<0.001*	2.60 (1.73–3.89)	<0.001*
Contact lens wear	0.59 (0.35–0.98)	<0.001*	0.50 (0.29–0.86)	**0.011**	0.50 (0.29–0.84)	**0.009**
LASIK/LASEK	0.97 (0.50–1.89)	0.940	0.95 (0.48–1.87)	0.875		
Cataract/RLE surgery	1.71 (0.81–3.62)	0.158	2.42 (1.08–5.41)	**0.031**	2.43 (1.10–5.39)	**0.028**
Glaucoma	1.29 (0.30–5.58)	0.734	1.68 (0.36–7.75)	0.509		
Antihistamines	1.36 (0.73–2.55)	0.335	1.27 (0.67–2.42)	0.469		
Proton‐pump inhibitors	2.30 (0.70–7.47)	0.168	2.64 (0.79–8.82)	0.114		
Isotretinoin	3.26 (0.44–24.3)	0.248	3.11 (0.41–23.6)	0.273		
Antidepressants	2.24 (0.80–6.26)	0.125	1.91 (0.67–5.45)	0.224		
Blepharoplasty	1.35 (0.52–3.46)	0.529	1.40 (0.53–3.73)	0.498		
Rosacea	4.08 (0.55–30.1)	0.168	3.02 (0.40–22.8)	0.285		
Hypothyroidism	3.13 (1.13–8.70)	**0.028**	2.62 (0.92–7.48)	0.072		

^a^ All the variables are dichotomous (0 and 1). *n* = 996 for all variables except glaucoma (*n* = 994) and rosacea (*n* = 995).

^b^ Multivariable analysis with all risk factors included (*n* = 993, pseudo‐*R* ^2^ = 0.097).

^c^ Multivariable analysis after backward stepwise selection (*n* = 996, pseudo‐*R* ^2y^ = 0.070).

^d^ *p*‐values <0.05 are shown in bold; those remaining significant after Bonferroni correction are marked with an asterisk (*).

^e^ Older age is defined as 65 years and above (≥65 years).

**FIGURE 2:**
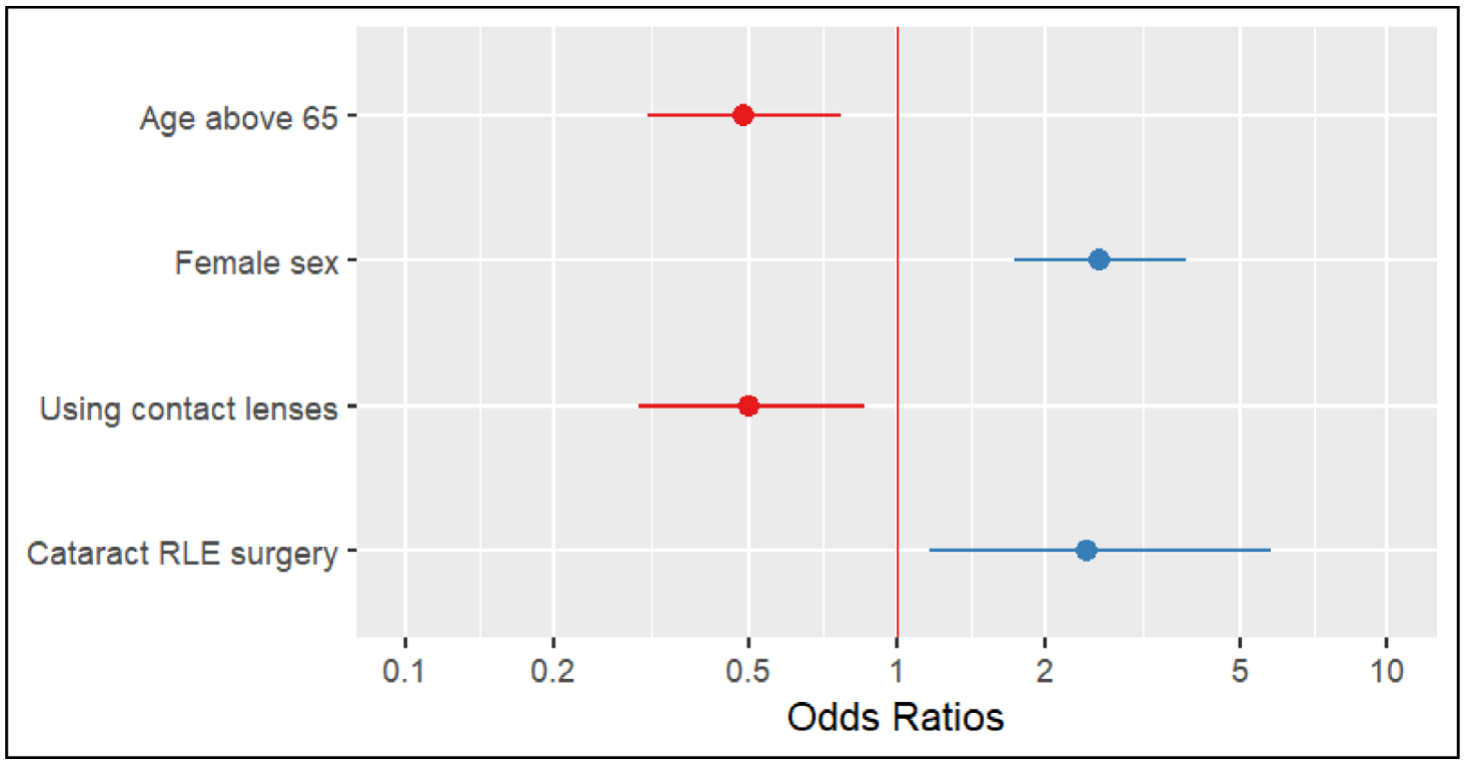
Forest plot depicting the final logistic regression model for factors associated with an OSDI score above the cut‐off value (≥13). The dots show the estimated odds ratios and the lines the corresponding 95% confidence intervals.

Other variables in Table 5, including LASIK/LASEK, cataract or refractive lens exchange (RLE) surgery, glaucoma, blepharoplasty, rosacea and medication use (antihistamines, proton‐pump inhibitors, isotretinoin and antidepressants), did not reach statistical significance. Notably, a few cases of keratoconus (*n* = 4), Sjögren's syndrome (*n* = 36), rheumatoid arthritis (*n* = 14) and systemic lupus erythematosus (*n* = 2) were found exclusively in the higher symptom severity OSDI groups, leading to incalculable ORs (data not shown). In multivariable analysis, adjusting for possible confounding among the factors age, sex and contact lens wear, the associations remained significant, with similar ORs as before and a lower *p*‐value for age. Furthermore, a borderline significant effect of RLE surgery was observed; however, it became non‐significant after Bonferroni correction (Table 5 and Figure 2).

## 4 DISCUSSION

The present study investigates the relationship between dry eye symptoms and sex, age and several other variables, as assessed by the OSDI and DEQ5 questionnaires (Table 1). A significant sex disparity was observed, with a markedly higher severity of DED symptoms among females compared to males, reinforcing the well‐documented influence of sex‐related factors on DED reported symptoms (Sullivan et al., 2017). The increasing prevalence of DED with age is well established (Stapleton et al., 2025). As the current study was conducted in a pre‐selected population rather than the general population, we did not aim to report prevalence numbers. In our dataset, DED symptoms above clinical cut‐off values were equally frequent in all age groups. Moreover, our data indicate that the younger patients from our cohort reported a higher degree of symptoms. Notably, a 2017 study (Vehof et al., 2017) examined patients in a tertiary dry eye clinic in the Netherlands (648 patients; mean age, 55.8 years; 82.7% female) who had been diagnosed with DED, yielding findings partly similar to ours. They reported that increased age and Sjögren syndrome were predictors of fewer dry eye symptoms, whereas in our study, age ≥ 65 years and contact lens use were associated with less severe symptom severity. In the Dutch study, however, the presence of allergy and the use of antihistamines were predictors of more symptoms, which we were unable to establish in our study. Notably, a tertiary clinic‐based study from Taiwan, including 1229 DED patients (79.3% female, mean age 56.7 years), found that female sex was a predictor of higher symptom severity, contrary to our findings, while older age was a predictor of lower symptom severity, consistent with our results (Lu et al., 2024). Collectively, these results underscore the importance of considering both biological and demographic factors in DED research.

The findings presented in Table 5 provide insight into factors associated with increased severity of dry eye symptoms among individuals presenting dry eye‐related complaints. Given the absence of a control group without dry eye symptoms, the analysis cannot be interpreted in terms of risk factors for developing dry eye per se. Instead, the data reflect associations with higher symptom‐reported burden, as defined by an OSDI score ≥ 13. The observed associations with sex and hypothyroidism are consistent with existing literature on hormones (Gorimanipalli et al., 2023), linking female sex (Sullivan et al., 2017) and thyroid dysfunction (Alanazi et al., 2019) to more pronounced dry eye symptoms. Interestingly, older age (≥65) and contact lens wear were associated with lower odds of reporting severe symptoms. In older individuals, peripheral neuropathy is more common and may reduce corneal sensitivity, potentially leading to fewer perceived symptoms of DED. Fewer symptoms in contact lens users may appear to contradict established knowledge that contact lens use is a risk factor for dry eye (Markoulli & Kolanu, 2017; Qian & Wei, 2022). However, it is essential to note that our cohort already comprises individuals with dry eye symptoms. Thus, the inverse association may reflect altered symptom perception, selection bias or behavioural modifications (e.g., discontinuation of lens use in more severely affected individuals). Dry eye symptoms may be perceived as more severe within this symptomatic cohort due to demographic and clinical modifiers. Interestingly, there are studies indicating that certain factors are predictors of discordance between dry eye symptoms and signs. For example, the presence of a known allergy, the use of allergy medications (antihistamines), depression and the use of anti‐depressants are predictors of more pronounced dry eye symptoms (Vehof et al., 2017), whereas older age and the use of artificial tears are among predictors of reduced symptoms (Lu et al., 2024; Vehof et al., 2017). Moreover, a cross‐sectional study on the relationship between dry eye symptoms and adiposity found that older age, body fat percentage, sex and contact lens wear were significant predictors of ocular discomfort (Ho et al., 2017). Collectively, these results underscore the importance of considering both biological and demographic factors in DED research.

The OSDI (cut‐off >13) and DEQ‐5 (cut‐off >6) are both validated questionnaires widely used in clinical and research settings for identifying dry eye symptoms and disease (Table 1) (Wolffsohn et al., 2017). Both questionnaires effectively screened and identified individuals in our large cohort, showing consistent utility in large‐scale studies. The OSDI provides a comprehensive assessment including environmental triggers and visual function, while the shorter DEQ‐5 focuses on symptom severity and frequency. Incorporating additional clinical tests may enhance diagnostic accuracy in individuals classified close to the diagnostic threshold (Wolffsohn et al., 2017). A limitation of both validated questionnaires is their inability to differentiate symptoms between the two eyes. The un‐validated in‐house questionnaire addresses the issue of laterality. However, it currently lacks a defined cut‐off value, limiting its clinical interpretability (Table S3). This questionnaire should be validated in future studies.

Patients with DED exhibit significant diurnal variation in visual function and clinical signs, with scores relatively high in the morning, decreasing in the late morning, increasing again in the afternoon and evening. This diurnal variation can be contributed to a gradual deterioration of the tear film and visual stability over the course of the day or a cumulative exacerbation of symptoms as visual tasks continue (Walker et al., 2010).

The reported time‐of‐day severity was highest in the morning and evening (Figure 1, Table 2). These findings align with reported diurnal variations in visual function and ocular surface physiology in individuals with dry eyes (Walker et al., 2010). The same study demonstrated greater evening visual impairment as measured by OSDI. Other studies on healthy adults also showed different patterns in the evening. For instance, levels of tear cytokines and chemokines (EGF, CX3CL1/fractalkine, CXCL10/IP‐10 and VEGF) were found to be higher in the evening compared to midday measurements (Benito et al., 2014). Additionally, the spontaneous eye‐blink rate increased in the evening (20:30 h), with further analysis suggesting an associated rise in central dopamine activity (Barbato et al., 2000). In the present study, the observed time‐dependent changes in symptom severity underscore the importance of accounting for time‐of‐day variability in dry eye assessments. These factors and recall bias may cause symptoms to be underestimated or overestimated depending on the timing of evaluations, potentially increasing the risk of false‐positive or false‐negative results and leading to erroneous clinical conclusions. These variations may be of clinical importance and merit consideration by the clinicians.

Among exploratory lifestyle findings, a few significant findings were observed in the different dietary groups (Table 4). Individual symptom analysis using the in‐house questionnaire revealed that vegans generally and vegetarians occasionally reported the lowest symptom severity scores. In contrast, pescatarians most frequently and vegetarians less frequently reported the highest values (Table S2). The lower scores among vegans and vegetarians may reflect the anti‐inflammatory nature of plant‐based diets (Menzel et al., 2020). Omega‐3 supplementation has, in a systematic review, been reported to improve DED symptoms (O'Byrne & O'Keeffe, 2023). Similarly, a meta‐analysis concluded that omega‐3 fatty acids effectively reduce DED symptoms, particularly at high doses, over prolonged periods and with increased eicosapentaenoic acid levels (Wang & Ko, 2023). In contrast, our findings indicate higher OSDI and DEQ‐5 scores in the omega‐3 supplementation group (Table 4), corresponding to increased symptom severity (Table S2). Although statistically significant, the differences in mean values between those taking and not taking omega‐3 were minimal, suggesting only minor effects. This may be attributed, in part, to the recruitment of individuals across varying severity levels without a healthy control group in the present study. Some individuals with more severe symptoms may be more inclined to take omega‐3 due to its perceived benefits, whilst others may have taken omega‐3 supplementation to manage or alleviate inflammatory or autoimmune disorders that are themselves linked to the development or worsening of DED. However, we did not find any association between omega‐3 supplementation and the recorded conditions related to inflammatory diseases, such as allergy, hypothyroidism, Sjögren's syndrome, as well as rheumatoid arthritis or systemic lupus erythematosus in our dataset (data not shown). Moreover, we cannot exclude other sources of high omega‐3 intake or the possibility of misinterpretation or reporting bias in the self‐administered questionnaires. Hence, the findings on diet and omega‐3 use and DED symptom severity reported herein should be interpreted with caution, and even more so due to the limited sample sizes of the dietary groups.

Although no significant association was found between the presence of allergies and overall dry eye symptoms (Table 4), specific symptoms such as redness and itching were significantly linked to allergies (Table S2). Notably, all parameters in the allergy group had higher mean values than those in the non‐allergy group across both tables. Our results differ from those of the Dutch study, which reported associations between the presence of allergy and symptom severity that contrast with our findings (Vehof et al., 2017). Ocular allergies are commonly associated with four hallmark symptoms, including eye redness, itching, swelling and excessive tearing (Rodrigues et al., 2021). Both ocular allergy and DED rank among the most prevalent disorders affecting the ocular surface, often occurring simultaneously and exhibiting considerable clinical overlap (Leonardi et al., 2021). Increasing evidence suggests that ocular allergy may play a contributing role in the development of DED (Villani et al., 2018). For instance, more than half of individuals diagnosed with ocular allergy are also reported to experience symptoms of dry eye. One proposed mechanism is that reduced tear production in dry eye may concentrate inflammatory mediators on the ocular surface (Rodrigues et al., 2021). Diagnosing DED becomes particularly complex when it coexists with, or is secondary to, ocular allergy (Rodrigues et al., 2021). Our results also showed that the difference between those with and without allergy was significant at all times of the day (Table 4), but the difference is most pronounced in the morning. This demonstrates the necessity of considering time‐of‐day variability in dry eye assessment.

One strength of the study is the large sample of patients with dry eye‐related symptoms, all examined at a single site by one of the two expert ophthalmologists, ensuring a high level of proficiency and consistency in diagnosing DED. At the same time, it is a limitation that all patients included in the NDEC2023 cohort sought care for ocular surface symptoms, resulting in selection bias and a higher prevalence of DED than in the general population. There may also be selection bias effects related to age, gender or other risk factors among individuals who seek care at the clinic. Consequently, the findings are not completely generalizable and should be interpreted within the context of this specific cohort. Additionally, the absence of a control group without DED symptoms prevents firm conclusions regarding causal risk factors, and the observed associations may reflect modifiers of symptom severity rather than true determinants. Moreover, the lack of objective ocular surface tests may limit the generalizability of symptom‐only outcomes. Symptom ratings were based on patient memory, which may have introduced recall bias and resulted in misclassifications. Furthermore, it should be acknowledged that some users may not have correctly ticked all relevant boxes and were therefore misclassified. Another limitation is the relatively small number of participants in certain subgroups, such as omega‐3 intake and diet, which may reflect underreporting. Finally, the use of an un‐validated in‐house questionnaire is a notable limitation, and the questionnaires used should be validated. While standardized timing of questionnaire administration could help minimize potential effects of time‐of‐day variation, such consistency is unfortunately generally impractical in both daily clinical practise and research settings due to logistical constraints.

## 5 CONCLUSIONS

We have investigated a battery of risk factors for DED in a clinic‐based study of dry eye patients. The findings may have clinical implications, as they collectively contribute to challenging some of the traditional assumptions in DED risk profiling. While contributors such as female sex, hypothyroidism and allergy are consistent with known risk profiles, the unexpected protective associations observed with age ≥ 65 and contact lens wear on symptom severity score highlight the need for an open‐minded approach and an in‐depth investigation. Overall, the findings underscore the importance of a more nuanced, individualized approach to DED evaluation and management. Future studies should validate the inter‐eye symptom questionnaire and examine these associations in population‐based cohorts to determine whether the observed patterns extend beyond symptomatic clinic populations.

## FUNDING INFORMATION

This research received no external funding.

## Supporting information


Appendix S1.
