# Supplementary material for: Dry eye disease symptoms and associated risk factors in a Norwegian clinical cohort
Source: Acta Ophthalmol. 2026 Apr 11;104(8):884–93. doi: 10.1111/aos.70143 (PMC13751863; doi:10.1111/aos.70143)
Supplement: Supplementary file 1 [file AOS-104-884-s001.docx]

**Supplementary Figure 1.** Histograms of the time-of-day variables.

**Supplementary Table 1.** Correlations between individual in-house symptom questions and patient-graded time of day with the symptoms. For each item, the first value is for the right eye and the second for the left, separated by a vertical dashed line in each box (Spearman correlation)

| *Spearman’s rho* | | **Gritty sensation** | | **Burning sensation** | | **Light sensitivity** | | **Stinging sensation** | | **Blurry vision** | | **Excessive tearing** | | **Red eye** | | **Itching** | | **Sticky eye** | |
| --- | --- | --- | --- | --- | --- | --- | --- | --- | --- | --- | --- | --- | --- | --- | --- | --- | --- | --- | --- |
|  |  |  |  |  |  |  |  |  |  |  |  |  |  |  |  |  |  |  |  |
| Time of day | Morning | 0.29 | 0.25 | 0.24 | 0.21 | 0.20 | 0.18 | 0.24 | 0.19 | 0.16 | 0.14 | 0.12 | 0.10 | 0.19 | 0.17 | 0.22 | 0.20 | 0.22 | 0.24 |
|  | Late morning | 0.29 | 0.29 | 0.32 | 0.30 | 0.25 | 0.25 | 0.25 | 0.22 | 0.20 | 0.18 | 0.15 | 0.14 | 0.16 | 0.15 | 0.21 | 0.22 | 0.12 | 0.15 |
|  | Afternoon | 0.28 | 0.30 | 0.32 | 0.32 | 0.27 | 0.26 | 0.22 | 0.21 | 0.23 | 0.22 | 0.13 | 0.12 | 0.17 | 0.16 | 0.23 | 0.24 | 0.07 | 0.10 |
|  | Evening | 0.36 | 0.33 | 0.30 | 0.29 | 0.29 | 0.26 | 0.23 | 0.21 | 0.26 | 0.23 | 0.07 | 0.06  **P = 0.062** | 0.20 | 0.18 | 0.25 | 0.24 | 0.12 | 0.14 |

^1^ Time of day was categorized as morning (before 09:00), late morning (09:00–12:00), afternoon (12:00–18:00), and evening (after 18:00)

All correlations were significant with p<0.001 (also significant after Bonferroni correction), unless stated otherwise (bold values)

Abbreviations: rho (Spearman’s rank correlation coefficient)

**Supplementary Table 2.** Associations between individual in-house symptom questions and other factors, including omega-3 supplementation, allergy presence and diet^1^ (Kruskal-Wallis test). Values for the left (OS) and right (OD) eyes are provided.

| **Right Eye (OD)** | **Omega-3** | | | **Allergy** | | | | **Diet** | | | | |
| --- | --- | --- | --- | --- | --- | --- | --- | --- | --- | --- | --- | --- |
|  | **Yes**  N: 501  Missing: 13  Median (IQR)  Mean ± SD | **No**  N: 488  Missing: 42  Median (IQR)  Mean ± SD | P-value^2^ | **Yes**  N: 299  Missing: 2  Median (IQR)  Mean ± SD | **No**  N: 690  Missing: 53  Median (IQR)  Mean ± SD | P-value^2^ | **Vegan**  N: 10  Missing: 1  Median (IQR)  Mean ± SD | | **Vegetarian**  N: 11  Missing: 0  Median (IQR)  Mean ± SD | **Pescatarian**  N: 17  Missing: 0  Median (IQR)  Mean ± SD | **Other**  N: 951  Missing: 54  Median (IQR)  Mean ± SD | P-value^2^ |
| **Gritty sensation** | 5 (1, 7)  4.65 ± 3.31 | 4 (0, 7)  4.08 ± 3.31 | **0.006** | 5 (1, 7)  4.48 ± 3.35 | 5 (1, 7)  4.32 ± 3.31 | 0.502 | 1.50 (0, 5.75)  2.90 ± 3.25 | | 4 (2.50, 7)  4.55 ± 2.98 | 1 (0, 6)  3.29 ± 3.74 | 5 (1, 7)  4.40 ± 3.31 | 0.286 |
| **Burning sensation** | 1 (0, 6)  2.79 ± 3.29 | 0 (0, 5)  2.31 ± 3.11 | **0.021** | 0 (0, 5)  2.58 ± 3.29 | 1 (0, 5)  2.54 ± 3.18 | 0.816 | 0 (0, 0)  1.00 ± 2.31 | | 2 (1, 7)  3.91 ± 3.51 | 2 (0, 7)  3.41 ± 3.55 | 1 (0, 5)  2.54 ± 3.20 | **0.037** |
| **Light sensitivity** | 4 (1, 7)  4.12 ± 3.32 | 3 (0, 6)  3.59 ± 3.23 | **0.009** | 4 (1, 7)  4.04 ± 3.33 | 4 (0, 7)  3.78 ± 3.27 | 0.250 | 0.50 (0, 3.25)  2.40 ± 3.72 | | 3 (0.50, 5)  3.09 ± 2.74 | 3 (1, 6)  4.00 ± 3.41 | 4 (0, 7)  3.88 ± 3.29 | 0.411 |
| **Stinging sensation** | 1 (0, 5)  2.40 ± 2.95 | 0 (0, 4)  2.08 ± 2.83 | **0.033** | 1 (0, 5)  2.44 ± 2.99 | 1 (0, 4)  2.15 ± 2.84 | 0.146 | 0 (0, 0.75)  1.10 ± 2.08 | | 2 (1, 4)  2.82 ± 2.36 | 1 (0, 4)  2.76 ± 3.31 | 1 (0, 4)  2.24 ± 2.90 | 0.237 |
| **Blurry vision** | 3 (0, 7)  3.78 ± 3.33 | 3 (0, 6)  3.26 ± 3.22 | **0.012** | 3 (0, 6)  3.69 ± 3.27 | 3 (0, 6)  3.46 ± 3.29 | 0.255 | 1 (0, 5.50)  2.80 ± 3.43 | | 3 (0.50, 4.50)  3.18 ± 3.34 | 5 (0, 7)  4.18 ± 3.49 | 3 (0, 6)  3.53 ± 3.28 | 0.728 |
| **Excessive tearing** | 2 (0, 6)  3.11 ± 3.41 | 2 (0, 6)  3.10 ± 3.45 | 0.967 | 2 (0, 6)  3.36 ± 3.51 | 1 (0, 6)  3.00 ± 3.39 | 0.168 | 1.50 (0, 2.75)  2.50 ± 3.34 | | 1 (0, 2)  1.27 ± 1.62 | 1 (0, 4)  2.82 ± 3.78 | 2 (0, 6)  3.14 ± 3.43 | 0.494 |
| **Red eye** | 3 (0, 6)  3.52 ± 3.26 | 2 (0, 5)  3.19 ± 3.16 | 0.109 | 3 (0, 6)  3.68 ± 3.25 | 2 (0, 6)  3.22 ± 3.19 | **0.039** | 2.50 (0, 3.75)  2.60 ± 2.91 | | 6 (0.50, 6.50)  4.09 ± 3.51 | 3 (0, 4)  2.94 ± 2.61 | 3 (0, 6)  3.37 ± 3.23 | 0.735 |
| **Itching** | 3 (0, 6)  3.31 ± 3.15 | 3 (0, 6)  3.31 ± 3.15 | 0.848 | 3 (0, 7)  3.72 ± 3.27 | 2 (0, 5)  3.13 ± 3.08 | **0.011** | 1 (0, 3.50)  2.40 ± 3.37 | | 1 (0.50, 3.50)  2.18 ± 2.27 | 5 (1, 7)  4.24 ± 3.15 | 3 (0, 6)  3.32 ± 3.15 | 0.311 |
| **Sticky eye** | 0 (0, 3)  1.77 ± 2.67 | 0 (0, 3)  1.72 ± 2.71 | 0.498 | 0 (0, 3)  1.94 ± 2.78 | 0 (0, 2)  1.67 ± 2.64 | 0.074 | 0 (0, 3.50)  1.80 ± 2.74 | | 0 (0, 1)  1.09 ± 2.39 | 0 (0, 3)  1.76 ± 2.66 | 0 (0, 3)  1.76 ± 2.69 | 0.881 |

| **Left Eye (OS)** | **Omega-3** | | | **Allergy** | | | **Diet** | | | | | |
| --- | --- | --- | --- | --- | --- | --- | --- | --- | --- | --- | --- | --- |
|  | **Yes**  N: 501  Missing: 13  Median (IQR)  Mean ± SD | **No**  N: 488  Missing: 42  Median (IQR)  Mean ± SD | P-value^2^ | **Yes**  N: 299  Missing: 2  Median (IQR)  Mean ± SD | **No**  N: 690  Missing: 53  Median (IQR)  Mean ± SD | P-value^2^ | **Vegan**  N: 10  Missing: 1  Median (IQR)  Mean ± SD | **Vegetarian**  N: 11  Missing: 0  Median (IQR)  Mean ± SD | **Pescatarian**  N: 17  Missing: 0  Median (IQR)  Mean ± SD | **Other**  N: 951  Missing: 54  Median (IQR)  Mean ± SD | P-value^2^ |  |
| **Gritty sensation** | 5 (2, 7)  4.71 ± 3.30 | 5 (1, 7)  4.24 ± 3.29 | **0.026** | 5 (1, 7.50)  4.75 ± 3.30 | 1 (0, 5)  2.58 ± 3.18 | 0.084 | 3 (0.25, 5)  3.10 ± 2.88 | 6 (3.50, 8)  5.27 ± 3.07 | 4 (1, 5)  3.94 ± 3.34 | 5 (1, 7)  4.49 ± 3.31 | 0.397 |  |
| **Burning sensation** | 1 (0, 5)  2.78 ± 3.28 | 0.50 (0, 5)  2.37 ± 3.13 | 0.051 | 1 (0, 5)  2.57 ± 3.29 | 1 (0, 5)  2.58 ± 3.18 | 0.612 | 0.00 (0, 0)  1.00 ± 2.31 | 6 (1, 7)  4.27 ± 3.50 | 2 (1, 7)  3.47 ± 3.32 | 1 (0, 5)  2.56 ± 3.21 | **0.014** |  |
| **Light sensitivity** | 4 (1, 7)  4.15 ± 3.32 | 3 (0, 6)  3.62 ± 3.26 | **0.008** | 4 (1, 7)  4.14 ± 3.34 | 4 (0, 7)  3.78 ± 3.28 | 0.103 | 0.50 (0, 3.25)  2.40 ± 3.72 | 3 (0.50, 6)  3.27 ± 2.87 | 5 (2, 8)  4.41 ± 3.41 | 4 (0, 7)  3.90 ± 3.30 | 0.359 |  |
| **Stinging sensation** | 1 (0, 5)  2.37 ± 2.96 | 0 (0, 4)  2.19 ± 2.94 | 0.139 | 1 (0, 5)  2.49 ± 3.02 | 1 (0, 4)  2.19 ± 2.91 | 0.112 | 0 (0, 0.75)  1.10 ± 2.08 | 2 (1, 4)  2.64 ± 2.29 | 3 (0, 7)  3.41 ± 3.36 | 1 (0, 4)  2.27 ± 2.95 | 0.108 |  |
| **Blurry vision** | 3 (0, 6)  3.73 ± 3.31 | 2 (0, 6)  3.23 ± 3.22 | **0.013** | 3 (0, 7)  3.81 ± 3.33 | 3 (0, 6)  3.35 ± 3.24 | **0.040** | 3.50 (0.50, 7.25)  3.80 ± 3.36 | 1 (0.50, 4.50)  3.09 ± 3.59 | 6 (2, 7)  4.94 ± 3.27 | 3 (0, 6)  3.46 ± 3.27 | 0.325 |  |
| **Excessive tearing** | 2 (0, 6)  3.10 ± 3.38 | 2 (0, 6)  3.17 ± 3.51 | 0.825 | 2 (0, 7)  3.44 ± 3.55 | 1 (0, 6)  3.01 ± 3.38 | 0.095 | 1.50 (0, 5)  2.80 ± 3.52 | 1 (0, 2)  1.27 ± 1.62 | 2 (0, 7)  3.35 ± 3.98 | 2 (0, 6)  3.16 ± 3.44 | 0.542 |  |
| **Red eye** | 3 (0, 6)  3.69 ± 3.28 | 3 (0, 6)  3.34 ± 3.15 | 0.102 | 4 (0, 6.50)  3.89 ± 3.29 | 3 (0, 6)  3.36 ± 3.18 | **0.027** | 3 (0, 4.75)  2.90 ± 3 | 6 (0.50, 6.50)  4.18 ± 3.66 | 4 (2, 4)  3.82 ± 2.81 | 3 (0, 6)  3.51 ± 3.23 | 0.774 |  |
| **Itching** | 3 (0, 6)  3.42 ± 3.19 | 3 (0, 6)  3.46 ± 3.19 | 0.978 | 4 (0, 7)  3.82 ± 3.25 | 3 (0, 6)  3.27 ± 3.15 | **0.019** | 1 (0, 4)  2.60 ± 3.41 | 2 (1, 5)  2.91 ± 2.74 | 6 (5, 7)  5.29 ± 2.78 | 3 (0, 6)  3.42 ± 3.19 | 0.069 |  |
| **Sticky eye** | 0 (0, 3)  1.94 ± 2.83 | 0 (0, 3)  1.83 ± 2.71 | 0.583 | 1 (0, 4)  2.08 ± 2.83 | 0 (0, 3)  1.80 ± 2.74 | 0.089 | 0 (0, 3.50)  1.80 ± 2.74 | 0 (0, 1)  0.91 ± 1.81 | 1 (0, 4)  2.18 ± 2.70 | 0 (0, 3)  1.89 ± 2.78 | 0.615 |  |

^1^ The diet has four categories

^2^ The p-values from the Kruskal-Wallis test for differences in distribution. P-values <0.05 are shown in bold; none remained significant after Bonferroni correction.

Abbreviations: Interquartile range (IQR), oculus dexter (OD), Oculus Sinister (OS), standard deviation (SD), sample size (N)

# Supplementary Table 3: In-house questionnaire developed by experts at the Norwegian Dry Eye Clinic.

Indicate the severity of any symptoms you have experienced since the last examination on a scale from 0 to 10, where 0 is no discomfort, 1 is very mild discomfort, and 10 is very severe discomfort (English version).

| **Symptoms** | **Right Eye (0–10)** | **Left Eye (0–10)** |
| --- | --- | --- |
| Gritty sensation |  |  |
| Burning sensation |  |  |
| Light sensitivity |  |  |
| Stinging sensation |  |  |
| Blurry vision |  |  |
| Excessive tearing |  |  |
| Red eye |  |  |
| Itching |  |  |
| Sticky eye |  |  |

What time of day are your symptoms most bothersome? Use the same grading scale as above.

| **Symptoms** | **Right Eye (0–10)** | **Left Eye (0–10)** |
| --- | --- | --- |
| Morning (before 9 AM) |  |  |
| Late morning (9 AM–12 PM) |  |  |
| Afternoon (12 PM–6 PM) |  |  |
| Evening (after 6 PM) |  |  |

Which season are your symptoms most bothersome? Use the same grading scale as above.

| **Symptoms** | **Right Eye (0–10)** | **Left Eye (0–10)** |
| --- | --- | --- |
| Spring |  |  |
| Summer |  |  |
| Autumn |  |  |
| Winter |  |  |

Diagnosed allergy?

| **Yes** |  |
| --- | --- |
| **No** |  |

Diet – check if applicable:

| **Omega-3 supplements (daily)** |  |
| --- | --- |
| **Vegan** |  |
| **Vegetarian** |  |
| **Pescetarian** |  |

How long have you had symptoms of dry eyes?

| Duration (indicate number of years or months) |  |
| --- | --- |

How has the condition developed since symptom onset? Check the most relevant options:

| Worse in connection with work | Gradual and steady worsening | Large variations |
| --- | --- | --- |
| Worse indoors | Worse outdoors | Little change over time |

Occupation: _________________________

Check the treatments you have tried. For current treatments, indicate the perceived effect on a scale from 0 to 10 (0 = no effect, 10 = very good effect).

| **Treatment** | **Check if applicable** | **Treatment effect (0–10)** |
| --- | --- | --- |
| Artificial tears |  |  |
| Eyelid hygiene/warm compress |  |  |
| Anti-inflammatory eye drops (e.g., Softacort, Monopex, Ikervis) |  |  |
| Antibiotics (e.g., chloramphenicol) |  |  |
| Punctal plugs |  |  |
| Light therapy |  |  |
| Other treatments received, Specify here: | | |

How many times per day do you use artificial tears in each eye (based on the last 7 days): ________________________

Some diseases are associated with dry eyes. Please list any relevant conditions:

Medications (both tablets and eye drops) can cause dry eyes. Please list the medications you use:

| **Tablets** | **Eye Drops** |
| --- | --- |
|  |  |
|  |  |
|  |  |

Finally, we want to assess your exposure to potential risk factors for dry eyes.

| **Possible Risk Factor** | **Checked if applicable** | **Approx. hours/day** |
| --- | --- | --- |
| Screen use |  |  |
| Contact lenses |  |  |
| Mascara |  |  |
| Eyeliner |  |  |
| Air-conditioning |  |  |

Thank you for taking the time to complete this questionnaire. Your responses will help us provide the best possible advice and guide treatment decisions.

Angi grad av eventuelle symptomer du har hatt siden forrige undersøkelse på en skala fra 0 til 10, hvor 0 er ingen plager, 1 er svært lette plager og 10 er svært uttalte plager (Norwegian version).

| **Symptomer** | **Høyre øye (0–10)** | **Venstre øye (0–10)** |
| --- | --- | --- |
| Sandfølelse |  |  |
| Brennende følelse |  |  |
| Lysømfintlighet |  |  |
| Stikkende fornemmelse |  |  |
| Varierende synsskarphet |  |  |
| Tåreflod |  |  |
| Rødt øye |  |  |
| Kløe |  |  |
| Sammenklistret øye |  |  |

Hvilket tidspunkt på døgnet er du mest plaget? Bruk samme gradering som ovenfor.

| **Symptomer** | **Høyre øye (0–10)** | **Venstre øye (0–10)** |
| --- | --- | --- |
| Morgen (før 9) |  |  |
| Formiddag (9–12) |  |  |
| Ettermiddag (12–18) |  |  |
| Kveld (etter 18) |  |  |

Hvilke(n) årstid er du mest plaget? Bruk samme gradering som ovenfor.

| **Symptomer** | **Høyre øye (0–10)** | **Venstre øye (0–10)** |
| --- | --- | --- |
| Vår |  |  |
| Sommer |  |  |
| Høst |  |  |
| Vinter |  |  |

Påvist allergi?

| **Ja** |  |
| --- | --- |
| **Nei** |  |

Kosthold - kryss av om aktuelt

| **Omega-3 tilskudd (daglig)** |  |
| --- | --- |
| **Veganer** |  |
| **Vegetarianer** |  |
| **Pescetarianer** |  |

Hvor lenge har du hatt symptomer på tørre øyne?

| Varighet (angi antall år, evt antall måneder) |  |
| --- | --- |

Hvordan har utviklingen vært siden symptomdebut? Kryss av på det eller de mest dekkende alternativene.

| Verst i forbindelse med jobb | Gradvis og jevn forverring | Store variasjoner |
| --- | --- | --- |
| Verst innendørs | Verst utendørs | Liten endring over tid |

Angi yrke: ________________________

Kryss av for de behandlinger du eventuelt har forsøkt. For aktuell behandling, angi opplevd effekt på en skala fra 0 til 10 (0 er ingen effekt, 10 er svært god effekt).

| **Behandling** | **Sett kryss om aktuelt** | **Behandlingseffekt**  **(0–10)** |
| --- | --- | --- |
| Kunstig tårevæske |  |  |
| Øyelokkrens/-varme |  |  |
| Betennelsesdempende øyedråper (f.eks Softacort, Monopex, Ikervis) |  |  |
| Antibiotika (f.eks kloramfenikol) |  |  |
| Tåreplugger |  |  |
| Lysbehandling |  |  |
| Om du har fått annet behandling, skriv dette her: | | |

Angi omtrent hvor mange ganger du drypper hvert øye med kunstig tårevæske per dag (ta utgangspunkt i de siste syv dagene): ________________________

Enkelte sykdommer er forbundet med tørre øyne. Om du har sykdommer, er det fint om de føres opp.

En rekke medisiner, både øyedråper og tabletter, kan resultere i tørre øyne. Vennligst før opp de medisiner du bruker:

| **Tabletter** | **Øyedråper** |
| --- | --- |
|  |  |
|  |  |
|  |  |

Avslutningsvis ønsker vi å avklare i hvilken grad du er blitt utsatt for mulige risikofaktorer for tørre øyne.

| **Mulige risikofaktor** | **Kryss av hvis aktuelt** | **Angi ca. antall timer per dag** |
| --- | --- | --- |
| Skjermbruk |  |  |
| Kontaktlinser |  |  |
| Mascara |  |  |
| Eyeliner |  |  |
| Air-condition |  |  |

Tusen takk for at du har tatt deg tid til å svare på spørsmålene. Svarene dine gjør det mulig for oss å gi deg best mulige råd og vil ha stor betydning for valg av behandling.
